# Influence Carrier Agents, Drying Methods, Storage Time on Physico-Chemical Properties and Bioactive Potential of Encapsulated Sea Buckthorn Juice Powders

**DOI:** 10.3390/molecules25173801

**Published:** 2020-08-21

**Authors:** Karolina Tkacz, Aneta Wojdyło, Anna Michalska-Ciechanowska, Igor Piotr Turkiewicz, Krzysztof Lech, Paulina Nowicka

**Affiliations:** 1 Department of Fruit, Vegetable and Plant Nutraceutical Technology, The Faculty of Biotechnology and Food Science, Wrocław University of Environmental and Life Sciences, 37 Chełmońskiego Street, 51-630 Wrocław, Poland; karolina.tkacz@upwr.edu.pl (K.T.); anna.michalska@upwr.edu.pl (A.M.-C.); igor.turkiewicz@upwr.edu.pl (I.P.T.); paulina.nowicka@upwr.edu.pl (P.N.); 2 Institute of Agricultural Engineering, Wrocław University of Environmental and Life Sciences, 37/41 Chełmońskiego Street, 51-630 Wrocław, Poland; krzysztof.lech@upwr.edu.pl

**Keywords:** *Hippophaë rhamnoides* L., inulin, maltodextrin, spray drying, freeze drying, vacuum drying, flavonols, HMF

## Abstract

Sea buckthorn (*Hippophaë rhamnoides* L.) juice with inulin, maltodextrin, and inulin:maltodextrin (1:2 and 2:1) were spray-, freeze- and vacuum-dried at 50, 70 and 90 °C. The study aimed to assess the impact of drying methods and carrier agents on physical properties (moisture content, water activity, true and bulk density, porosity, color parameters, browning index), chemical components (hydroxymethylfurfural and phenolic compounds) and antioxidant capacity of sea buckthorn juice powders. Storage of powders was carried out for six months. Inulin caused stronger water retention in powders than maltodextrin. Vacuum drying provided powders with the highest bulk density. Maltodextrin did not promote browning and HMF formation as strongly as inulin. More phenolic compounds were found in powders with maltodextrin. Storage increased the antioxidant capacity of powders. The results obtained will be useful in optimizing the powders production on an industrial scale, designing attractive food ingredients.

## 1. Introduction

Sea buckthorn (*Hippophaë rhamnoides* L.) belongs to the Elaeagnaceae family and occurs mainly in the northern hemisphere. The high content of flavonols, L-ascorbic acid and lipophilic compounds including carotenoids, tocopherols, fatty acids and phytosterols provides unique health-promoting properties and thus enables a wide range of applications of this plant [1,2]. Juices, beverages, jams, oils, teas, pharmaceuticals, cosmetics, dairy and spirits as well as feedstuff are produced from sea buckthorn fruits, leaves, bark and seeds. To date, anti-radical activity, protection against UV radiation, efficacy in dermatological diseases, cardioprotective, hepatoprotective, anti-inflammatory, anti-hyperlipidemic, anti-cholinergic, anti-hypertensive, anti-hyperinsulinemia and antimicrobial properties have been studied [2,3]. Due to the high fat content, liquid and semi-liquid products from sea buckthorn separate into two phases and thus are not attractive to consumers. An alternative can therefore be the process of juice encapsulation using drying methods leading to the formation of powders. However, drying pure fruit juices is hindered due to agglomeration of material particles and adhesion to the surface of dryer installations. Fruit juices, including sea buckthorn, contain organic acids and sugars with a low glass transition temperature (Tg). Therefore, the high-molecular weight carrier agents are mixed with the juices before drying to increase the Tg of the product and, as a consequence, avoid a viscoplastic state and caking [4,5]. The carrier agents used in the production of powders may be maltodextrin, inulin, gum arabic, carrageenan gum, carboxymethyl cellulose (CMC), starch, pectin, whey protein, gelatin, casein and others; however, each of them affects the physical and chemical properties of products [6,7,8]. The most commonly used techniques are spray drying and freeze drying. However, the potential for vacuum drying, drum drying, reactance window drying, microwave-vacuum and other combined drying is increasing [4,6,9].

Powders from whole fruits, juice, extract and pomace are produced, depending on the form of the fruit. For instance, powders from mango [10], apple juice [11], Roselle extract [8], orange juice with incorporated lactic acid bacteria [12], grape skin phenolic extract [13], purple sweet potato [6], grape wastes [14], pomegranate peel phenolics [15], blackberry phenolics [16], herb extract [7] and with probiotics in raspberry juice [9] have been produced and studied thus far.

Encapsulation involves entrapment of valuable, sensitive or target components or fractions within the coating material. Processing fruit juice into powder can extend its shelf life and thus improve its physical properties and nutritional and pro-healthy value, as in the research by Bąkowska-Barczak and Kołodziejczyk [17], Aziz et al. [4] and Çam et al. [15]. The development of sea buckthorn powders may facilitate the potential use of health benefits of sea buckthorn with their prolonged shelf life and lower transport and storage costs. The encapsulated juice form offers flexibility for innovative formulas and uses as a replacement for juices and concentrates and in new markets, including bakery products, confectionery, sauces, ice cream, dairy and nutritional and functional snacks. Juice powders can fit well with the trend of using natural thickeners and agents that change or enhance the taste, color and health value of products. Additionally, reducing the instability of sea buckthorn bioactive compounds during processing and storage, as well as digestion in the digestive system, may meet the expectations of the cosmetics and pharmaceutical industries [6,8,10]. Thus, the formation of sea buckthorn juice powders can be equally beneficial.

This study aimed to assess the impact of drying methods (spray drying, freeze drying and vacuum drying at 50, 70 and 90 °C) and types of carrier agents (inulin, maltodextrin and mixtures inulin:maltodextrin in the ratio of 1:2 and 2:1) on physical properties (moisture content, water activity, true and bulk density, porosity, color parameters, browning index), chemical components (hydroxymethylfurfural [HMF] and phenolic compounds) and antioxidant capacity of sea buckthorn juice powders before and after six-month storage. To the best of our knowledge, this is the first detailed report on powders from *H. rhamnoides* juice. It will provide valuable information on the selection of carrier agents and optimal drying conditions, stability of chemical compounds and antioxidant activity of sea buckthorn juice after drying processes and then after storage.

## 2. Results and Discussion

### 2.1. Physical Properties of Sea Buckthorn Juice Powders

**Figure 1:**
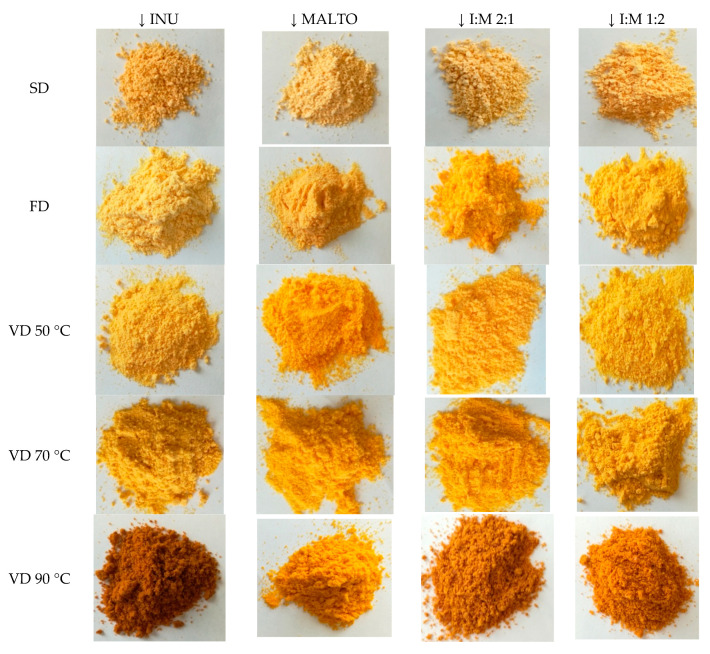
Color of sea buckthorn juice powders modulated by drying methods and carrier agents. SD, spray drying; FD, freeze drying; VD, vacuum drying; INU, inulin; MALTO, maltodextrin; I:M, inulin:maltodextrin.

Physical properties, such as moisture content, water activity, true and bulk density and porosity of sea buckthorn juice powders are summarized in Table 1. Color parameters, chroma parameter, the total color change, hue angle, and browning index are presented in Table 2. Color of sea buckthorn juice powders are presented on Figure 1. Tested properties can be used in processing control, quality of the final product and its storage stability, but also for estimation of the texture of powders [4,18].

#### 2.1.1. Moisture Content

The moisture content of sea buckthorn juice powders ranged from 1.29% (vacuum-dried powder at 90 °C with inulin:maltodextrin 1:2) to 4.96% (vacuum-dried powder at 50 °C with inulin) (Table 1). All powders met the moisture criterion below 5% for microbiological safety [4]. Nevertheless, the kind of carrier agents, the drying methods and their parameters modulated the moisture content. The moisture-differentiating factors could be temperature during freeze drying (too low cause sublimation barrier) [13], inlet and outlet temperatures during spray drying and percentage of carrier agents [19]. Selvamuthukumaran and Khanum [20] found that the inlet air temperature, followed by maltodextrin concentration, had the maximum effect on the moisture content of the spray-dried sea buckthorn juice. In the same research, they also found that the optimal values of these parameters were, respectively, 162.5 °C and 1:4 for maltodextrin:fruit slurry. Thus, the determination of water sorption isotherms for powders can be extremely helpful in modeling drying, conditioning and storage processes. The sorption of water by sugar is weaker at higher temperatures, therefore research could be directed towards the effect of the degree of maltodextrin dextrinization causing an increase in water absorption and sugar content in plant material and powders [21].

In this study, the water content for sea buckthorn juice powders after freeze drying and vacuum drying at 50 °C was comparable moisture (approximately 3.51% and 3.78%, respectively). Higher temperatures (spray drying at 180 °C similar to vacuum drying at 90 °C) significantly reduced the moisture content (approximately 2.12% and 1.54%, respectively). Similar observations were obtained by Bąkowska-Barczak and Kołodziejczyk [17] in the research on encapsulated blackcurrant polyphenols; however, our study also revealed differences between powders with maltodextrin and inulin. The addition of inulin caused stronger water retention than in the case of maltodextrin powders (approximately 3.71% and 2.01%, respectively), which can be explained by the high hygroscopicity of inulin resulting from the branched structure that promotes hydrogen bonding and moisture absorption from ambient air [22]. The influence of maltodextrin on moisture content is ambiguous, as dextrinization causes an increase in the number of ramifications with hydrophilic groups, but lower DE was associated with better binder properties [23]. Nevertheless, other studies also confirm lower moisture content in powders containing maltodextrin, compared to other carrier agents, e.g., gum arabic, corn starch, pectin, carboxymethylcellulose and yucca starch [8,24].

The results obtained for sea buckthorn juice powders are typical of the average moisture content of previously created powders, including blackcurrant polyphenol extracts (1.8–3.9%) [17], chokeberry powders (1.13–4.05%) [25], spray-dried cinnamon infusions with maltodextrin (1.34–1.99%) [21] and spray-dried grape skin phenolic extract (2.41–2.57%) [13].

#### 2.1.2. Water Activity

The water activity of sea buckthorn juice powders ranged from 0.074 (vacuum-dried powder containing maltodextrin) to 0.101 (freeze-dried powder containing inulin) (Table 1). Maintaining the water activity of powders at the level obtained will prevent or minimize the growth of mold, yeast and bacteria, degradation of biologically active compounds and non-enzymatic browning.

The type of drying method caused greater variation in the water activity than the type of carrier agents. The water activity of powders increases with equilibrium moisture content at a constant temperature [26], therefore the lowest water activity was characterized by powders vacuum-dried at 90 °C (approximately 0.075). The values for powders after freeze drying and vacuum drying at 50 °C was the same (approximately 0.098), and similar with that for powders vacuum-dried at 70 °C (approximately 0.093). Freeze-dried powders may have higher water activity than those after other methods due to their higher porosity which facilitates water penetration in the pores [11]. Additionally, the nozzle diameter and feed flow rate in spray drying [23,24], and structure and content of carrier agents (higher concentration causes a decrease in the a_w_) [19] could modulate the a_w_ in powders. Statistically similar water activity was observed for powders with inulin and with inulin:maltodextrin (1:2) (approximately 0.092), as well as those with maltodextrin and with inulin:maltodextrin (2:1) (0.089 and 0.088, respectively).

The water activity results obtained were similar or lower than those for probiotic orange powders (0.34–0.42) [12], spray-dried watermelon powders (0.20–0.29) [19], microencapsulated Andes berry extracts (0.199–0.422) [24], spray-died microencapsulated betalains cactus (0.176–0.205) [27], microencapsulated Bordo grape skin extract (0.160–0.360) [13] and cantaloupe juice powders (0.15–0.19) [28].

#### 2.1.3. True and Bulk Density

The values of true density for sea buckthorn juice powders ranged between 1240 (spray-dried powder with maltodextrin) and 1543 kg m^−3^ (freeze-dried powder with inulin) (Table 1). Generally, spray dried particles have lower true density values than freeze dried and vacuum dried products [11]. The same relationship was found for sea buckthorn powders, which can be ranked according to decreasing true density values as follows: freeze-dried > vacuum-dried at 50 and 70 °C > vacuum-dried at 90 °C > spray-dried powders (approximately 1519, 1479, 1408 and 1374 kg m^−3^, respectively). The largest diversity in true density values was recorded for spray-dried powders (1240–1471 kg m^−3^), whereas powders containing inulin had significantly higher true density values than those with maltodextrin (approximately 1472 and 1423 kg m^−3^, respectively). As expected, true density of all powders was higher than true density of corresponding pure carrier agents. Furthermore, the largest increase in true density was determined for powder with maltodextrin after freeze drying (difference of approximately 300 kg m^−3^).

Bulk density values did not correlate with true density (r = 0.187). The lowest bulk density values were determined for freeze-dried powder with inulin:maltodextrin (1:2) and spray-dried powder with maltodextrin (373.1 and 389.5 kg m^−3^, respectively). For this first powder, the largest difference between its bulk density and bulk density of the pure carrier agent (575.4 kg m^−3^) was also calculated. Sea buckthorn juice powder with inulin:maltodextrin (2:1), vacuum-dried at 90 °C, had the highest bulk density (597.1 kg m^−3^) and this value did not differ significantly from the bulk density of the analogous pure carrier agent (547.5 kg m^−3^). Moreover, extremely bulk density values were observed for powders with blend of inulin and maltodextrin 1:2 and 2:1, respectively, compared to powders with simple carrier agents. This differentiation was explained by polymer interactions between carbohydrates and juice powder in the study by Ferrari et al. [26]. Powders encapsulated with maltodextrin had a minor bulk density than that produced with a mix of gum arabic and maltodextrin.

Bulk density and porosity depend on particle size, inter-particle voids of powders, properties of juice and carrier agents, drying methods and temperatures [10]. Particles with higher moisture amount tend to have a higher bulking weight due to the presence of water which is denser than dried solids [26]. However, in this study, no correlation was found between moisture content and bulk density (r = −0.183) and weak correlation between moisture content and porosity (r = 0.406). In addition, sea buckthorn fruits contain low sugar levels (average 2%), so the powder obtained by spray drying was not sticky. Drying methods had a larger impact on bulk density than carrier agents. Thus, powders in terms of decreasing bulk density can be ordered as follows: vacuum-dried at 90 °C > vacuum-dried at 50 and 70 °C > freeze-dried > spray-dried powder (approximately 561.6, 538.1, 461.3 and 451.2 kg m^−3^, respectively). This behavior can be explained by the crystal structure of the powders obtained in a vacuum and by the reduction of interstitial air content between the particles and the reduction of the volume occupied [11,26].

High bulk density is economically positive, since products require smaller packaging, and transport and storage costs are lower. Lower bulk density is associated with entrapment of air in voids, thereby facilitating oxidation and less stability [4]. Pure inulin had a higher value than maltodextrin (644.3 and 472.3 kg m^−3^, respectively) due to its molecular weight and thus heavier material and limited spaces between particles. Nevertheless, the bulk density of sea buckthorn juice powders with inulin (approximately 514.5 kg m^−3^) decreased relative to the pure carrier agent, but with maltodextrin favorably increased (mean 512.7 kg m^−3^). The studied powders had similar bulk density values to those determined for spray-dried cinnamon infusions with maltodextrin (536–554 kg m^−3^) [21] and spray-dried Roselle extract with maltodextrin, pectin, carboxymethylcellulose, gum arabic and whey powder (427–588 kg m^−3^) [8]. However, this second research team investigated higher differentiation, with double the bulk density for the powder with carrageenan gum (849 kg m^−3^) than for gelatin (392 kg m^−3^).

#### 2.1.4. Porosity

The drying methods had a greater impact on the powder porosity than the type of carrier agents. Measured porosity values were between 57.71% (powder with inulin:maltodextrin in the ratio of 2:1 after vacuum drying at 90 °C) and 75.43% (freeze-dried powder with inulin:maltodextrin 1:2). The porosity of sea buckthorn juice powders obtained by vacuum drying at 50 and 70 °C was similar and averaged 62.47%. Consequently, in terms of porosity, powders can be ranked as follows: freeze-dried (approximately 69.63%) ≈ spray-dried (approximately 67.19%) > vacuum-dried (between approximately 60.12% and 63.74%). Based on scanning electron micrographs, Azizpour et al. [29] stated that the high temperature promotes the increase in porosity of dried material. However, other studies suggested that surface roughness is typical for spray dried powders at low temperatures. Such a structure promotes higher humidity and suppleness, and, consequently, it causes a reduced volume of particles during cooling [22,23].

The porosity of the pure carrier agents differed significantly, ranging between 53.53% for inulin and 61.65% for maltodextrin. Despite this, the porosity of the sea buckthorn juice powders with inulin was higher by less than 1% compared to those with maltodextrin (64.97% and 64.09%, respectively). Increased porosity in powders with maltodextrin and thus a significant reduction in bulk density was investigated for spray-dried mango puree [10]. In turn, the smoother surface of the insulin particles can be ascribed to the relatively low polydispersity and higher molecular flexibility enabling many conformations. An increase in temperature and a low concentration of this oligosaccharide in final powders (20%) may have contributed to a decrease in its crystallinity and viscosity [30].

The largest variation in the porosity was found between powders produced by freeze drying. Reduced temperature and pressure in this process ensure an appropriate sublimation rate; the material is not exposed to shrinkage, and, as a result, products with high porosity and rehydration capacity are created [10]. Thus, the porosity and bulk density of sea buckthorn juice powders may be an important criterion of application, due to the storage conditions, the type and form of the final product and oxidative and aromatic stability.

#### 2.1.5. Color Parameters

The color parameters of sea buckthorn juice powders are given in Table 2, while images, showing clear differences in color, are presented in Figure 1.The color of the powders was determined with reference to the CIE L*a*b* color space, in which parameter L* indicates brightness from blackness (0) to whiteness (100), parameter a* determines the color from green (−) to red (+) and parameter b* is from blue (−) to yellow (+). In the sea buckthorn juice powders, coordinate L* ranged from 54.55 (vacuum-dried powder with inulin:maltodextrin in the ratio of 2:1) to 89.26 (spray-dried powder with maltodextrin). Powders produced by freeze drying and spray drying had higher parameter L* values than those after vacuum drying (especially in higher temperature variants). In previous studies, similar trends were obtained, which indicates the beneficial use of these drying processes in the production of powders with favorable, minimally changed color. Prolonged exposure to 90 °C under vacuum could have promoted browning reactions such as caramelization and Maillard reactions [12,25].

Moreover, the powders after vacuum drying at 90 °C were the darkest (L* = approximately 59.28) but the powder with maltodextrin had a significantly higher parameter L* equal to 71.52. Such a large difference between the brightness of powders with maltodextrin and the remaining powders was found only in this drying method. Other researchers have also described higher parameter L* after the addition of maltodextrin in raspberry powders, orange juice powders and mango powders [9,10,12]. The superiority of maltodextrin over insulin in the context of final brightness may find corroboration in various interactions between juice matrix and carrier agents and, consequently, the release of compounds that undergo further reactions.

The hue angle (h°), which characterizes the perception of color, showed values from 43.29° (vacuum dried powder at 90 °C with inulin:maltodextrin 2:1) to 91.88° (sprayed powder with maltodextrin), thus indicating values between red (0°) and yellow (90°). For spray-dried powders, followed by freeze-dried and vacuum-dried at 50 and 70 °C, the highest h° values were recorded, i.e. the closest to the angle for yellow. The type of carrier agent did not significantly affect h° but the parameters a* and b* were significantly different for powders with inulin, maltodextrin and their mixtures. The lowest h° values were recorded for powders dried in vacuum at 90 °C, which could still be caused by browning reactions. Coordinate b* indicates a more yellow color of the powders with maltodextrin than those with inulin (b* = approximately 52.05 and 43.75, respectively), however the h° values indicate such results only for drying techniques at higher temperatures (spray drying and vacuum drying at 90 °C).

The chroma parameter (C), indicating the purity and intensity of color, of the sea buckthorn juice powders ranged from 35.80 (powder with inulin after vacuum drying at 90 °C) to 59.89 (powder with maltodextrin after vacuum drying at 70 °C). The highest intensity of color was characterized by powders with maltodextrin, and the lowest with inulin (C = approximately 55.72 and 44.25, respectively). The average values of the parameter C for powders after freeze drying and vacuum drying at 50 and 70 °C did not differ significantly. The color intensity of sea buckthorn powders obtained by spray drying was the lowest, as was the case with orange, mango and apple powders [10,11,12]. Nevertheless, opposite results were obtained by Kuck and Noreña [13], and thus higher parameter C values were observed for spray-dried grape skin extracts than after freeze drying. The reason may be the use of gum arabic, polydextrose and guar gum as encapsulating agents. The values of the chroma parameter strongly correlated with the value of parameter b* (r = 0.922) and poorly with the parameter a* (r = 0.218), indicating a strong yellow color of powders.

The total color change (dE) was calculated using sea buckthorn juice as a reference (L* = 60.44; a* = 24.84; b* = 51.75). It was found that the color change resulted from the parameter L* increase and the parameter a* decrease in the powders obtained. Thus, the dE ranged between 17.30 (powder with inulin:maltodextrin 1:2, after vacuum drying at 90 °C) to 41.64 (spray-dried powder with maltodextrin). All results indicate visible and distinguishable differences in the color of powders for the human eye (dE > 5.0) [22] but do not suggest explicit sensory assessments of color.

Drying methods had a stronger impact on the color change than the type of carrier agents. The highest average dE was observed for spray-dried powders, and the lowest after vacuum drying 90 °C (approximately 38.96 and 20.47, respectively). Powders with maltodextrin were characterized by the smallest color change (dE = approximately 27.03), again suggesting the appropriateness of this carrier agent to produce sea buckthorn powders.

#### 2.1.6. Browning Index

The browning index was presented as an indicator of non-enzymatic browning of powders. In powders before storage, the browning index ranged from 0.09 AU (powder with maltodextrin after vacuum drying at 70 °C) to 0.71 AU (powder with inulin after vacuum drying at 90 °C). Freeze drying and vacuum drying at 50 °C resulted in the lowest browning of powders, in contrast to vacuum drying at 90 °C (approximately 0.16, 0.18 and 0.43 AU, respectively). Inulin promoted browning reactions, so powders can be ranked according to the increasing browning index: powders with maltodextrin < powders with inulin:maltodextrin 1:2 and 2:1 < powders with inulin.

Research on model systems has proved that browning reactions at temperatures above 60 °C occur significantly more quickly in a model containing glucose than in one containing galactose. Glucose is the dominant sugar in sea buckthorn; hence, the long-term vacuum drying at 90 °C resulted in the highest browning index. In addition, the browning index could be modulated by carrier agents, drying temperature, storage process, content of sugars and compounds with a free amino group in powders [31]. Generally, inulin is non-reducing and does not contain or form reactive ketone and aldehyde groups. However, it is a polydisperse mixture and may contain more reactive mono- and disaccharides and as a consequence may participate in reactions with groups of other compounds, including in Maillard reactions. Importantly, low pH and elevated temperature may promote its hydrolysis in aqueous solutions, thereby increasing the amount of reducing sugars. This should be particularly taken into account when using sea buckthorn juice with a naturally low pH about 3.0 [30].

After six months of storage, the browning index varied between 0.50 AU (spray-dried powder with maltodextrin) and 2.98 AU (vacuum-dried powder at 90 °C with inulin). Powders vacuum-dried at 90 °C had the highest browning index (approximately 2.53 AU), and simultaneously almost six times higher compared to powders before storage. As with fresh powders, stored powders with inulin had the highest average index, and the lowest with maltodextrin (approximately 1.24 and 0.79 AU).

### 2.2. Hydroxymethylfurfural in Sea Buckthorn Juice Powders

The hydroxymethylfurfural (HMF) content was tested in powders before and after six months of storage (Table 3). In fresh powders, the HMF content ranged from 0.05 (powder with inulin:maltodextrin 2:1 after vacuum drying at 70 °C) to 75.21 mg/100 g DM (powder with inulin after vacuum drying at 90 °C). The average HMF content in powders spray-dried, freeze-dried and vacuum-dried at 50 °C did not differ significantly and ranged between 0.15 and 0.94 mg/100 g DM. Moßhammer et al. [32] also reported that cactus pear powders after freeze drying and spray drying had low and similar HMF concentration.

The temperature increase during vacuum drying significantly increased the amount of HMF formed, and thus powders vacuum-dried at 90 °C had 47.12–75.21 mg/100 g DM, except for that with maltodextrin (0.39 mg/100 g DM). Similarly, Michalska et al. [33] reported that the HMF formation was slow at temperatures above 60 °C, and rapid above 80 °C, during convection drying of blackcurrant pomace powders.

The average HMF content in powders with maltodextrin was 28–45 times lower than in powders with other carrier agents (approximately between 11.56 and 18.16 mg/100 g DM). This indicates the appropriateness of using pure maltodextrin for the production of powders by the vacuum method at higher temperatures. The results obtained were in line with the research on plum powders, in which maltodextrin also impeded HMF creation [34]. It should be noted, however, that short-term spray drying at 180 °C did not result in high HMF concentration regardless of carrier agents.

HMF is one of the products of the advanced Maillard reaction, occurring in products with hexose, as a result of thermal treatment and storage, or a degradation product of ascorbic acid. Therefore, in stored powders, the HMF amount increased and equaled 0.15–101.40 mg/100 g DM (powder with inulin:maltodextrin 2:1 after freeze drying and after vacuum drying at 90 °C, respectively). Powders spray-, freeze- and vacuum-dried at 50 °C had significantly similar average HMF content, similar as in fresh powders (approximately between 0.33 and 1.57 mg/100 g DM).

However, the increase in HMF after storage was significantly lower for spray-dried powders. This could be due to the formation of spherical capsules in spray drying, without pores and thus with full core protection [35]. The increase in HMF after storage was the highest in the case of powders with maltodextrin (almost 12 times), whereas the content of this compound was the lowest in comparison to powders with other carrier agents (4.80 vs. 17.77–29.45 mg/100 g DM). In addition, the correlation between HMF content and browning index was higher for powders after storage than before (r = 0.970 and 0.669).

### 2.3. Phenolic Compounds in Sea Buckthorn Juice Powders

The content of phenolic compounds before and after six months of storage is presented in Table 3. Sea buckthorn berries are a rich source of flavonols, which account for over 98% of the total phenolic compounds, as proven in previous studies [2]. Similarly, in the case of powders, flavonols were also the dominant phenolic compounds. The greatest variation in their content was tested within powders vacuum-dried at 90 °C (between 181.80 for powders with inulin:maltodextrin 1:2 and 298.68 mg/100 g DM for powders with maltodextrin). Vacuum drying at 70 °C had the most beneficial effect on retaining sea buckthorn flavonols (approximately 271.71 mg/100 g DM). In terms of the type of carrier agents, powders can be ranked according to decreasing flavonol concentration: powders with: maltodextrin > with inulin:maltodextrin 1:2 and 2:1 > with inulin (from 267.35 to 215.38 mg/100 g DM). Flavonols in stored powders were found in concentrations from 144.05 (spray-dried powder with inulin:maltodextrin 1:2) to 256.46 mg/100 g DM (vacuum-dried powder at 70 °C with maltodextrin). Flavonol degradation after freeze drying was the lowest (by 4.8%) and thus their concentration in these powders was the highest (approximately 119.76 mg/100 g DM). The lowest amount of flavonols was determined in inulin powders, and their reduction after six months was the smallest (by 10.6%). The flexibility of the inulin skeleton combined with high glass transition temperature (Tg) make this agent a proper stabilizer of nutritional and bioactive components. For example, in food and pharmaceutical applications it is a suitable stabilizer of proteins in the dry state [30]. Therefore, the results could be strongly dependent on the retention degree of polyphenols and their stability in the powders encapsulated with carrier agents which display different protection characteristics and kinetic parameters.

Phenolic acid content was from 0.24 (powder vacuum-dried at 70 °C with inulin:maltodextrin 2:1 and vacuum-dried powder at 90 °C with inulin:maltodextrin 1:2) to 3.00 mg/100 g DM (spray-dried powder with maltodextrin). Spray-dried powders contained on average 3.5 times more phenolic acids than powders vacuum-dried at 90 °C (2.51 and 0.71 mg/100 g DM, respectively). The results obtained were in line with those obtained by Horszwald et al. [25], who reported that spray drying caused the least degradation of flavonoids in chokeberry powders compared to freeze drying and vacuum drying. The highest average phenolic acid content (1.83 mg/100 g DM) was found in powders with maltodextrin. Stored powders contained from 0.10 (vacuum-dried powder at 90 °C with inulin:maltodextrin 1:2) to 2.96 mg phenolic acids/100 g DM (spray-dried powder with maltodextrin). Six-month storage resulted in stronger degradation of phenolic acids in powders after vacuum drying at 90 °C (by 50.7%) than after freeze drying (by 2.8%). The highest concentration of phenolic acids (approximately 1.65 mg/100 g DM) was measured in powders with maltodextrin, and the degradation of these compounds (by 9.8%) was the lowest compared to powders with other carrier agents (up to 18.5%).

The contents of flavonols and phenolic acids in pure sea buckthorn juice were 578.30 and 5.25 mg/100 g DM, respectively. Thus, the powders contained on average 2–3 times less phenolic compounds immediately after drying and 2–4 times less after the storage process compared to pure juice. The loss of phenolic compounds after drying may result from the use of high temperatures, exposure to oxygen, formation of fissures, concavities, microspheres and pores, which cause release and degradation of the encapsulated component [13]. The sea buckthorn powders were stored in the presence of oxygen and moisture, which favor the phenol compound degradation reactions and changes in the structure of the carrier agents [36]. Moreover, flavonol concentration correlated with porosity of sea buckthorn powders with different agents (r = 0.913), which may explain the easier release of polyphenols in extraction process, as well as their lower retention during storage and thus exposure to degradation. On the other hand, previous research on stored blackcurrant microcapsules obtained by spray drying showed that inulin created a more stable product with polyphenols than maltodextrins, whereas the stability of polyphenols in powders with maltodextrin was dependent on dextrose equivalent (DE) and those with DE 11 provided greater protection for polyphenols during storage than with DE 18 and DE 21 [17]. Higher degree of carrier polymerization results in lower retention of compounds present in the encapsulated material due to the sensitivity of shorter carrier carbohydrate units to temperature and thus their deformation [36]. In research on spray-dried Roselle extract, Díaz-Bandera et al. [8] observed that maltodextrin, similar to carrageenan gum, carboxymethyl cellulose, gelatin and gum arabic, showed low polyphenol release values at the steady state. Mensink et al. [30] emphasized, however, that both the mean DP and actual size distribution of carrier agents determining rheological and thermal properties should be taken into account.

### 2.4. Antioxidant Capacity of Sea Buckthorn Juice Powders

Antioxidant capacity of sea buckthorn juice powders estimated by the ABTS·^+^ method ranged from 0.85 (powders vacuum-dried at 90 °C with inulin:maltodextrin 1:2) to 1.73 mmol Trolox/100 g DM (powders freeze-dried with inulin). Powders obtained by spray, freeze and vacuum drying at 50 °C had the highest antioxidant capacity (approximately 1.55 mmol Trolox/100 g DM). The average antioxidant effects of powders, except for powders with inulin and maltodextrin (1:2), were similar and ranged from 1.41 to 1.55 mmol Trolox/100 g DM.

Higher antioxidant capacity was measured for powders stored for six months than for fresh powders. An almost 50% increase in antioxidant capacity was found for powders dried at 90 °C under vacuum, but they showed the lowest activity (1.75 mmol Trolox/100 g DM). The increase in antioxidant capacity of spray- and freeze-dried powders averaged 26.4%. Similar to research on blackcurrant polyphenol microcapsules [17], the antioxidant capacity of powders with inulin was the most stable after storage, but powders with maltodextrin showed stronger activity towards the cation radicals.

Spray drying had the most favorable effect on preserving the antioxidant capacity of powders. There was only 4.6 times less antioxidant activity than for pure juice (7.53 mmol Trolox/100 g DM), compared to almost nine times difference for powders obtained by vacuum drying at 90 °C. However, as noted by Santiago-Adame et al. [21], the activity of products dried by this method is conditioned by parameters, and 180 °C and feed rate at 10 mL/min are the most desired. Browning compounds did not affect the increase in antioxidant capacity, and there was also no correlation between changes in antioxidant activity and content of flavonols and phenolic acids.

**Figure 2:**
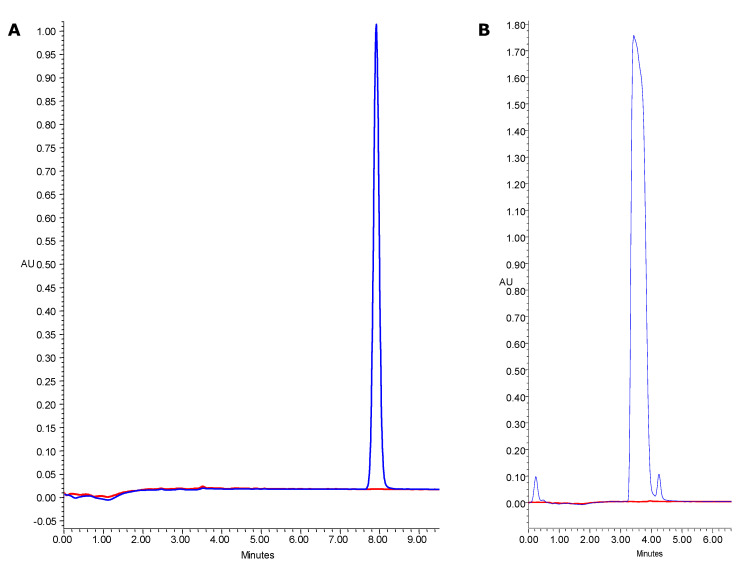
Chromatographic profile HPLC-PDA obtained before and after the derivatization using the ABTS·^+^ reagent for: hydroxymethylfurfural (**A**); and furosine (**B**). The measurement was performed three times for independent samples and there were no significant differences between the results. (*p* > 0.05).

On-line profiling was conducted to verify the potential antioxidant capacity of HMF and furosine. Furosine is an early product of the Maillard reaction and, similar to HMF, is formed during high temperature processing and storage. Figure 2 shows the chromatographic HMF and furosine profile obtained before and after the derivatization process with ABTS·^+^ reagent serving as a negative control. The upper chromatogram refers to the absorbance at 280 nm, and the lower one is the response after the reaction with ABTS·**^+^** reagent at 734 nm. The absence of negative responses after the post-column reaction suggests that HMF and furosine had no radical scavenging capacity. Moreover, there was no correlation between the HMF content and the increase in antioxidant effect. In research on blackcurrant pomace powders, Michalska et al. [33] also concluded that compounds with antioxidant capacity are not formed during the Maillard reaction.

To explain the increase in antioxidant capacity, further analysis of powder structure and other compounds found in sea buckthorn powders and the kinetics of their degradation or formation should be performed. Analysis of phenolic and other compounds by LC-MS in powders before and after storage can be valuable. According to Rocha-Parra et al. [37], losses of phenolic compounds in freeze-dried encapsulated red wine were also not reflected in antioxidant capacity changes. Reactions between oxidized phenolics can therefore increase the antioxidant capacity.

## 3. Materials and Methods

### 3.1. Chemicals

All standards used for Ultra-Performance Liquid Chromatography Photodiode Array Detector (UPLC-PDA) assays were bought from Extrasynthese (Lyon, France). Ascorbic acid and acetonitrile for ultraperformance liquid chromatography UPLC (gradient grade), carrier agents and the rest of the reagents were procured from Merck (Darmstadt, Germany).

### 3.2. Material and Sample Preparation

Sea buckthorn berries of cultivar “Józef” were collected from the Experimental Orchard in Dąbrowice of the Research Institute of Horticulture in Skierniewice (Poland). Sea buckthorn juice was squeezed from selected fruits using a laboratory hydraulic press (SRSE, Warsaw, Poland), centrifuged at 5000× *g* for 10 min (Sigma 6 K15, Shrewsbury, UK) and portioned into four parts. Each portion was mixed with 20% (*w/w*) commercial inulin (INU), maltodextrin (MALTO) and inulin with maltodextrin in 2:1 (I:M) and 1:2 proportions (I:M), separately. The 20% addition of carrier agents was determined experimentally on the basis of solubility in juice, drying tests and the properties of finished products.

### 3.3. Drying Methods

Each variant of the juice with carrier agent was divided into five parts (ca. 100 mL each) to undergo various drying methods: spray drying (SD), freeze drying (FD) and vacuum drying (VD) at three different temperatures. The spray drying process of the sea buckthorn juices with different carrier agents was performed using a Bϋchi Mini Spray-Dryer B-290 (Bϋchi AG, Flawil, Switzerland). The initial temperature of the juices was 21 °C. The spray dryer operated at an inlet temperature of 180 °C and the feeding rate was 40 mL min^−1^. The freeze-drying process was performed at temperatures from −30 to +30 °C, a pressure of 0.22 mbar and for 24 h using a Christ Alpha 1–4 LSC (Martin Christ GmbH; Osterode am Harz, Germany). The choice of carrier agents and drying parameters was determined experimentally. The drying time was determined on the basis of previous drying tests for juices in the temperature range of 50–90 °C using the determination of water. The water content was determined on the basis of the mass losses of samples during drying in a previous drying test. The process was stopped when the moisture content of samples reached below 5%. The vacuum drying processes at temperatures of 50, 70, and 90 °C were done using a Vacucell ECO line (MMM Medcenter Einrichtungen GmbH, Planegg/München, Germany), at a pressure below 0.1 mbar for 24, 20 and 16 h, respectively. The conditions used in the three drying methods were adequate to ensure complete drying of the samples with a final moisture content below 5%. All drying processes were performed in triplicate. The sea buckthorn juice powders obtained (Figure 1) were vacuum-sealed in transparent polyamide/polyethylene (PA/PE) moisture-resistant bags and stored at −18 °C for further analyses. Physical analyses and sample extractions for chemical analyses and evaluation of antioxidant activity were performed within 5 days from the production of the powders.

### 3.4. Storage

To determine the potential progress of the browning reaction, HMF and phenolic compounds contents, and the antioxidant activity of powders, a storage test was carried out. The second batch of sea buckthorn juice powders was stored in transparent polyamide/polyethylene (PA/PE) bags, for six months, at 20 °C and relative humidity 40%, with access to oxygen, in darkness. A laboratory incubator (ST2, POL-EKO-APARATURA, Wodzisław Śl.; Poland) was used to maintain stable conditions. After this period, the powders were again subjected to selected analyses.

### 3.5. Physical Properties

Moisture content (%) of the sea buckthorn juice powders was determined by the vacuum-oven method at 70 °C and pressure of 100 Pa for 24 h, using the vacuum dryer from Section 3.3. Water activity (a_w_) was studied at 20 °C using a dedicated device a Novasina (LabMaster-aw, Lachen, Switzerland). True and bulk density (kg m^−3^) and porosity (%) were studied and calculated as previously described by Turkiewicz et al. [38]. Color parameters were measured using a spectrophotometer Minolta Chrome Meter CM-700d (Konica Minolta, Inc.; Osaka, Japan) and expressed in scale of CIE L*a*b* space (10°, D65). Chroma parameter (C), hue angle (h°) and the total color change (dE) were calculated according to Kuck and Noreña [13] and Šumić et al. [39]. Browning index was determined in powder extracts (1 g of powder in 100 mL of distilled water). The results were measured at 420 nm using a multi-mode microplate reader Synergy^TM^ H1 (BioTek, Winooski, VT, USA) and shown in arbitrary units (AU).

### 3.6. Determination of Phenolic Compounds and Hydroxymethylfurfural (HMF)

Analysis of phenolic compounds and hydroxymethylfurfural (HMF) were performed using an Ultra-Performance Liquid Chromatography with Photodiode Array Detector (UPLC-PDA, Acquity UPLC System, Waters Corp.; Milford, WA, USA). The extraction procedure and analysis conditions of phenolic compounds and HMF were analogous to those given previously by Tkacz et al. [2] and Turkiewicz et al. [40], respectively. Quantification was made on the basis of standard curves, using HMF; *p*-coumaric and ferulic acids; 3-*O*-glucosides, 3-*O*-rutinosides and 3-*O*-rhamnosides of isorhamnetin; quercetin; and kaempferol as standards. The other flavonol derivatives were calculated as the corresponding 3-*O*-glucoside derivatives. Phenolic acids, flavonols and HMF were detected at wavelengths 320, 360 and 284 nm, respectively. The results were expressed as mg per 100 g of dry matter (DM).

### 3.7. Determination of Antioxidant Capacity and Antioxidant On-Line Profiling by HPLC-PDA Coupled with Post-Column Derivatization with ABTS·^+^ Reagent

The antioxidant capacity was tested as free radical-scavenging activity (ABTS·**^+^**). The extraction and assay were conducted as previously described by Tkacz et al. [41]. The multi-mode microplate reader discussed in Section 3.5 was used. The results of antioxidant effects were calculated as mmol Trolox/100 g DM.

An on-line HPLC system was applied to verify the possible antioxidant capacity of HMF and furosine. The same ABTS·**^+^** reagent was used as in antioxidant capacity assay. Conditions and procedure of the assay were analogous as reported by Tkacz et al. [42]. The detection wavelengths for HMF and furosine were set at 280 nm, and discoloration of mobile phase after reaction with radical cation was detected as negative peaks at 734 nm. The chromatograms are shown as results.

### 3.8. Statistical Analysis

One-way analysis of variance (ANOVA) with a significance below 0.05, Duncan’s multiple range test and Pearson’s correlation coefficients (r) were determined to compare the samples. XLSTAT Statistical Software (Addinsoft Inc, New York, NY, USA) integrated with Microsoft Excel 2017 (Microsoft Corp.; Redmond, WA, USA) were used. Drying tests were performed three times and replicates were samples from each trial. Each of the analyses was performed three times and the results were summarized in the form of the mean with standard deviation (SD).

## 4. Conclusions

For the first time, research was conducted on the optimization of microencapsulation of sea buckthorn juice using both different drying methods and different carrier agents. The main results of this paper can be summarized as follows:

(1) Inulin caused stronger water retention of powders than maltodextrin. The drying method modulated the water activity more strongly than the type of carrier agents.
(2) Powders with inulin had higher true density values than those with maltodextrin. Bulk density and porosity were significantly differentiated by drying methods, and vacuum drying seems to be a useful technique to obtain powders with high bulk density. The porosity of the spray-dried and freeze-dried powders was higher than after vacuum drying.
(3) In view of the yellow color and its intensity, the use of maltodextrin was competitive compared to inulin. Moreover, spray-, freeze- and vacuum-drying at 50 °C and the addition of maltodextrin were not conducive to browning and HMF formation.
(4) Powders spray- and vacuum-dried at 70 °C had the highest concentrations of phenolic acids and flavonols, respectively. However, in stored freeze-dried powders, phenolic compound losses were the lowest. More phenolic compounds were determined in powders with maltodextrin.
(5) Storage for six months increased antioxidant capacity, but browning compounds, HMF and furosine did not affect this effect.

In conclusion, the results obtained will be useful in the selection of carrier agents and optimization of drying conditions on an industrial scale. Encapsulation technique can be valuable for extending the stability of sea buckthorn juice and for designing innovative and high-quality products, such as attractive functional foods or food ingredients, improving physical and health-promoting properties. The choice of carrier agent and its interaction with the juice should be further investigated to ensure minimal degradation of biologically active compounds and beneficial properties of finished powders. In the future, it will also be valuable to study the stability, bioavailability and kinetics of biologically active compounds released from powders or real food systems by in vitro and in vivo methods.

## Figures and Tables

**Table 1:** Moisture content, water activity, true density, bulk density and porosity of sea buckthorn juice powders.

Drying Method	Carrier Agent	Moisture Content (%)	Water Activity (a_w_)	True Density (kg m^−3^)	Bulk Density (kg m^−3^)	Porosity (%)
SD	INU	2.62 ± 0.11 d	0.086 ± 0.001 d	1408 ± 15 c	488.2 ± 13 d	65.34 ± 0.6 d
	MALTO	2.01 ± 0.16 de	0.090 ± 0.002 cd	1240 ± 9 e	389.5 ± 5 f	68.52 ± 0.1 c
	I:M 2:1	1.64 ± 0.10 e	0.080 ± 0.001 d	1375 ± 13 d	459.6 ± 23 de	66.59 ± 1.3 d
	I:M 1:2	2.19 ± 0.12 de	0.089 ± 0.000 cd	1471 ± 12 b	466.5 ± 10 de	68.29 ± 0.4 c
FD	INU	4.75 ± 0.18 b	0.101 ± 0.001 c	1543 ± 10 a	448.1 ± 4 e	70.95 ± 0.1 b
	MALTO	2.55 ± 0.12 d	0.096 ± 0.000 c	1529 ± 13 ab	548.9 ± 18 bc	64.10 ± 0.9 d
	I:M 2:1	3.28 ± 0.14 c	0.097 ± 0.001 c	1485 ± 15 b	474.9 ± 11 d	68.03 ± 0.4 c
	I:M 1:2	3.45 ± 0.16 c	0.098 ± 0.001 c	1519 ± 8 ab	373.1 ± 3 f	75.43 ± 0.1 a
VD 50 °C	INU	4.96 ± 0.20 ab	0.099 ± 0.001 c	1508 ± 11 ab	541.9 ± 12 bc	64.08 ± 0.5 d
	MALTO	2.42 ± 0.10 d	0.096 ± 0.001 c	1485 ± 13 b	550.5 ± 18 bc	62.94 ± 0.9 de
	I:M 2:1	3.68 ± 0.13 c	0.097 ± 0.000 c	1480 ± 15 b	541.0 ± 19 bc	63.45 ± 0.9 d
	I:M 1:2	4.04 ± 0.11 bc	0.100 ± 0.001 c	1462 ± 12 b	519.1 ± 11 c	64.49 ± 0.5 d
VD 70 °C	INU	4.31 ± 0.13 b	0.098 ± 0.000 c	1479 ± 10 b	542.7 ± 12 bc	63.30 ± 0.6 d
	MALTO	1.69 ± 0.15 e	0.088 ± 0.002 d	1467 ± 9 b	549.4 ± 9 bc	62.57 ± 0.4 de
	I:M 2:1	1.99 ± 0.18 de	0.088 ± 0.001 d	1473 ± 12 b	539.9 ± 21 bc	63.35 ± 1.1 d
	I:M 1:2	3.98 ± 0.22 bc	0.096 ± 0.001 c	1475 ± 13 b	515.9 ± 6 c	65.03 ± 0.1 d
VD 90 °C	INU	1.89 ± 0.11 e	0.076 ± 0.000 e	1421 ± 11 c	551.7 ± 3 bc	61.19 ± 0.1 e
	MALTO	1.36 ± 0.17 ef	0.074 ± 0.000 e	1393 ± 14 cd	524.5 ± 5 c	62.34 ± 0.1 de
	I:M 2:1	1.62 ± 0.12 e	0.076± 0.002 e	1412 ± 11 c	597.1 ± 8 b	57.71 ± 0.3 f
	I:M 1:2	1.29 ± 0.19 f	0.075 ± 0.002 e	1406 ± 12 c	573.1 ± 15 b	59.23 ± 0.7 e
Pure carrier agents	INU	2.08 ± 0.01 de	0.129 ± 0.001 b	1387 ± 11 d	644.3 ± 9 a	53.53 ± 0.3 h
	MALTO	5.70 ± 0.25 a	0.397 ± 0.001 a	1228 ± 12 ef	472.3 ± 2 d	61.65 ± 0.2 e
	I:M 2:1	5.07 ± 0.00 ab	0.383 ± 0.001 a	1273 ± 13 e	547.5 ± 5 bc	57.00 ± 0.1 f
	I:M 1:2	4.65 ± 0.08 b	0.368 ± 0.001 a	1308 ± 9 f	575.4 ± 6 b	56.01 ± 0.2 g
**Duncan’s Multiple Range Test**						
Drying method	SD	2.12 C	0.086 B	1374 D	451.2 D	67.19 A
	FD	3.51 A	0.098 A	1519 A	461.3 C	69.63 A
	VD 50 °C	3.78 A	0.098 A	1484 B	538.1 B	63.74 B
	VD 70 °C	2.99 B	0.093 AB	1474 B	537.0 B	63.56 B
	VD 90 °C	1.54 D	0.075 C	1408 C	561.6 A	60.12 C
Carrier agent	INU	3.71 A	0.092 A	1472 A	514.5 B	64.97 AB
	MALTO	2.01 D	0.089 B	1423 D	512.7 B	64.09 B
	I:M 2:1	2.44 C	0.088 B	1445 C	522.5 A	63.83 B
	I:M 1:2	2.99 B	0.092 A	1467 B	489.6 C	66.49 A

Data are shown as mean (*n* = 3) ± standard deviation; for each parameter tested, values with different letters differ significantly (Duncan’s test, *p* < 0.05); SD–spray drying; FD, freeze drying; VD, vacuum drying; INU, inulin; MALTO, maltodextrin; I:M, inulin:maltodextrin.

**Table 2:** Color parameters and browning index of sea buckthorn juice powders.

Drying Method	Carrier Agent	Color Parameters						Browning Index (AU)	
		L*	a*	b*	Chroma (C)	dE	Hue Angle (h°)	0 Months	6 Months
SD	INU	86.57 ± 0.14 bc	1.56 ± 0.03 g	40.81 ± 0.08 d	40.84 ± 0.38 d	36.67 ± 0.13 c	87.81 ± 0.04 d	0.22 ± 0.00 de	0.74 ± 0.02 e
	MALTO	89.26 ± 0.17 b	−1.21 ± 0.05 i	36.77 ± 0.05 e	36.79 ± 0.41 e	41.64 ± 0.16 b	91.88 ± 0.06 c	0.24 ± 0.01 d	0.50 ± 0.01 g
	I:M 2:1	88.53 ± 0.09 b	−0.63 ± 0.11 i	38.76 ± 0.02 de	38.77 ± 0.67 de	40.08 ± 0.08 b	90.93 ± 0.13 c	0.43 ± 0.02 b	0.58 ± 0.01 f
	I:M 1:2	87.12 ± 0.16 bc	0.97 ± 0.05 h	40.70 ± 0.08 d	40.71 ± 0.65 d	37.47 ± 0.15 c	88.64 ± 0.06 d	0.28 ± 0.01 cd	0.59 ± 0.01 f
FD	INU	87.58 ± 0.17 bc	−0.36 ± 0.23 hi	46.67 ± 0.05 c	46.67 ± 0.44 c	37.38 ± 0.15 c	90.44 ± 0.24 c	0.30 ± 0.02 c	0.72 ± 0.01 e
	MALTO	81.99 ± 0.04 c	6.32 ± 0.06 d	50.16 ± 0.06 bc	50.56 ± 0.37 b	28.46 ± 0.03 e	82.82 ± 0.07 g	0.16 ± 0.00 e	0.52 ± 0.01 fg
	I:M 2:1	78.87 ± 0.06 d	9.66 ± 0.04 c	57.43 ± 0.02 ab	58.24 ± 0.20 a	24.55 ± 0.05 f	80.45 ± 0.05 g	0.14 ± 0.01 e	0.59 ± 0.01 f
	I:M 1:2	83.79 ± 0.19 c	3.55 ± 0.03 ef	55.24 ± 0.07 b	55.35 ± 0.85 ab	31.79 ± 0.16 d	86.32 ± 0.04 de	0.11 ± 0.01 ef	0.47 ± 0.01 g
VD 50 °C	INU	84.59 ± 0.20 bc	1.64 ± 0.10 g	47.89 ± 0.06 c	47.92 ± 0.34 c	33.71 ± 0.18 d	88.04 ± 0.12 d	0.19 ± 0.01 e	0.61 ± 0.01 f
	MALTO	77.00 ± 0.09 d	11.98 ± 0.02 bc	55.53 ± 0.03 b	56.81 ± 0.40 ab	21.31 ± 0.07 fg	77.83 ± 0.04 h	0.15 ± 0.02 e	0.64 ± 0.01 f
	I:M 2:1	80.99 ± 0.03 c	6.05 ± 0.05 d	53.57 ± 0.02 b	53.91 ± 0.12 b	27.91 ± 0.01 e	83.57 ± 0.06 f	0.11 ± 0.01 ef	0.66 ± 0.01 f
	I:M 1:2	82.88 ± 0.28 c	2.96 ± 0.04 f	53.70 ± 0.04 b	53.78 ± 0.54 b	31.40 ± 0.24 d	86.85 ± 0.05 de	0.18 ± 0.01 e	0.55 ± 0.01 fg
VD 70 °C	INU	77.48 ± 0.03 d	4.62 ± 0.04 e	50.32 ± 0.02 bc	50.53 ± 0.79 b	26.48 ± 0.01 ef	84.75 ± 0.05 f	0.17 ± 0.01 e	1.16 ± 0.01 d
	MALTO	77.59 ± 0.17 d	9.12 ± 0.06 c	59.19 ± 0.03 a	59.89 ± 0.98 a	24.43 ± 0.15 f	81.24 ± 0.07 g	0.09 ± 0.00 f	0.70 ± 0.01 ef
	I:M 2:1	77.64 ± 0.04 d	9.19 ± 0.06 c	58.86 ± 0.10 a	59.57 ± 0.11 a	24.32 ± 0.03 f	81.13 ± 0.07 g	0.32 ± 0.02 c	0.74 ± 0.01 e
	I:M 1:2	77.62 ± 0.14 d	4.56 ± 0.05 e	52.87 ± 0.05 b	53.07 ± 0.31 b	26.61 ± 0.13 ef	85.57 ± 0.06 e	0.25 ± 0.01 d	1.00 ± 0.01 d
VD 90 °C	INU	54.85 ± 0.09 f	12.03 ± 0.07 b	33.06 ± 0.05 f	35.80 ± 0.69 e	23.34 ± 0.08 f	70.00 ± 0.08 i	0.71 ± 0.02 a	2.98 ± 0.03 a
	MALTO	71.52 ± 0.03 e	10.57 ± 0.08 bc	58.60 ± 0.01 a	59.55 ± 0.87 a	19.33 ± 0.03 g	79.78 ± 0.08 g	0.28 ± 0.01 cd	1.57 ± 0.01 c
	I:M 2:1	54.55 ± 0.04 f	35.78 ± 0.04 a	33.70 ± 0.09 f	49.15 ± 0.83 b	21.91 ± 0.03 fg	43.29 ± 0.09 k	0.30 ± 0.01 c	2.84 ± 0.03 ab
	I:M 1:2	56.21 ± 0.12 f	13.26 ± 0.07 b	39.62 ± 0.07 d	41.78 ± 0.69 d	17.30 ± 0.10 h	71.50 ± 0.08 i	0.42 ± 0.01 b	2.72 ± 0.01 b
Pure carrier agents	INU	97.58 ± 0.01 a	−1.51 ± 0.01 i	4.53 ± 0.01 g	4.78 ± 0.01 f	65.60 ± 0.01 a	108.44 ± 0.01 b	-	-
	MALTO	98.03 ± 0.00 a	−1.24 ± 0.01 i	2.74 ± 0.01 h	3.01 ± 0.01 f	67.05 ± 0.01 a	114.35 ± 0.01 a	-	-
	I:M 2:1	97.90 ± 0.01 a	1.47 ± 0.01 i	3.75 ± 0.01 gh	4.03 ± 0.01 f	65.22 ± 0.01 a	68.60 ± 0.02 ij	-	-
	I:M 1:2	98.02 ± 0.00 a	1.37 ± 0.01 i	3.33 ± 0.01 gh	3.60 ± 0.01 f	65.63 ± 0.01 a	67.64 ± 0.01 j	-	-
**Duncan’s Multiple Range Test**									
Drying method	SD	87.87 A	0.17 C	39.26 D	39.28 C	38.96 A	89.82 A	0.29 B	0.60 C
	FD	83.06 AB	4.79 B	52.38 B	52.70 A	30.55 B	85.01 B	0.18 CD	0.58 C
	VD 50 °C	81.37 B	5.66 B	52.67 B	53.10 A	28.58 B	84.07 BC	0.16 D	0.62 C
	VD 70 °C	77.58 C	6.87 B	55.31 A	55.76 A	25.46 C	83.17 C	0.21 C	0.90 B
	VD 90 °C	59.28 D	17.91 A	41.25 C	46.41 B	20.47 D	66.14 D	0.43 A	2.53 A
Carrier agents	INU	78.21 AB	3.90 D	43.75 C	44.23 D	31.52 A	70.17 A	0.32 A	1.24 A
	MALTO	79.47 A	7.36 B	52.05 A	55.72 A	27.03 C	68.93 A	0.18 C	0.79 C
	I:M 2:1	76.12 C	12.01 A	48.46 B	51.93 B	27.75 BC	62.23 B	0.26 B	1.08 B
	I:M 1:2	77.52 BC	5.06 C	48.43 B	48.94 C	28.91 B	69.81 A	0.25 B	1.07 B

Data are shown as mean (*n* = 3) ± standard deviation; for each parameter tested, values with different letters differ significantly (Duncan’s test, *p* < 0.05); SD–spray drying; FD, freeze drying; VD, vacuum drying; INU, inulin; MALTO, maltodextrin; I:M, inulin:maltodextrin; dE, total color change; AU, arbitrary units.

**Table 3:** Content of hydroxymethylfurfural (HMF) and phenolic compounds (mg/100 g DM) and antioxidant capacity (mmol Trolox/100 g DM) of sea buckthorn juice powders.

Drying Method	Carrier Agent	HMF		Phenolic Acids		Flavonols		Antioxidant Capacity	
		0 Months	6 Months	0 Months	6 Months	0 Months	6 Months	0 Months	6 Months
SD	INU	1.50 ± 0.13 e	1.79 ± 0.19 f	2.86 ± 0.11 ab	2.71 ± 0.15 a	210.83 ± 4.05 f	203.92 ± 2.23 e	1.62 ± 0.08 ab	2.25 ± 0.23 c
	MALTO	0.39 ± 0.09 g	0.72 ± 0.11 g	3.00 ± 0.29 a	2.96 ± 0.19 a	222.21 ± 2.45 e	209.51 ± 2.46 e	1.56 ± 0.08 b	1.43 ± 0.19 ef
	I:M 2:1	0.82 ± 0.10 f	1.89 ± 0.20 f	2.49 ± 0.09 b	2.26 ± 0.13 b	242.05 ± 5.17 d	174.96 ± 2.01 f	1.60 ± 0.02 ab	2.20 ± 0.03 c
	I:M 1:2	1.05 ± 0.02 ef	1.88 ± 0.18 f	1.70 ± 0.14 c	1.26 ± 0.10 de	266.43 ± 3.55 b	144.05 ± 2.12 g	1.64 ± 0.10 ab	2.27 ± 0.22 c
FD	INU	0.04 ± 0.01 i	0.67 ± 0.14 gh	1.71 ± 0.10 c	1.67 ± 0.21 d	213.28 ± 4.44 ef	200.40 ± 2.32 ef	1.73 ± 0.19 a	2.10 ± 0.34 cd
	MALTO	0.41 ± 0.10 g	0.57 ± 0.11 h	1.85 ± 0.24 bc	1.80 ± 0.14 cd	251.48 ± 4.64 c	249.02 ± 3.54 ab	1.45 ± 0.20 c	2.09 ± 0.12 cd
	I:M 2:1	0.07 ± 0.01 i	0.15 ± 0.08 i	1.44 ± 0.18 d	1.37 ± 0.24 d	255.78 ± 2.47 c	233.09 ± 3.53 c	1.56 ± 0.07 b	1.85 ± 0.22 d
	I:M 1:2	0.09 ± 0.01 i	0.17 ± 0.10 i	2.12 ± 0.22 b	2.07 ± 0.07 c	245.40 ± 3.89 d	236.53 ± 2.79 c	1.40 ± 0.07 c	1.72 ± 0.29de
VD 50 °C	INU	0.45 ± 0.06 g	0.51 ± 0.10 h	1.39 ± 0.16 d	1.00 ± 0.25 e	217.69 ± 2.56 e	150.42 ± 3.72 g	1.56 ± 0.08 b	1.80 ± 0.34 d
	MALTO	0.44 ± 0.03 g	0.50 ± 0.07 h	1.57 ± 0.20 cd	1.49 ± 0.24 d	274.25 ± 3.52 b	256.46 ± 2.64 a	1.46 ± 0.11 c	1.96 ± 0.17 d
	I:M 2:1	0.13 ± 0.01 i	0.89 ± 0.22 g	1.60 ± 0.15 cd	1.23 ± 0.17 de	248.55 ± 3.12 cd	222.51 ± 2.70 d	1.55 ± 0.14 b	2.61 ± 0.17 b
	I:M 1:2	0.32 ± 0.09 h	0.90 ± 0.06 g	1.02 ± 0.09 e	0.92 ± 0.15 e	273.93 ± 2.25 b	223.91 ± 3.75 d	1.46 ± 0.05 c	2.35 ± 0.08 bc
VD 70 °C	INU	14.09 ± 1.53 c	21.12 ± 2.14 c	1.21 ± 0.13 de	0.94 ± 0.13 e	243.86 ± 2.51 d	212.50 ± 2.34 e	1.40 ± 0.18 c	1.65 ± 0.20 e
	MALTO	0.46 ± 0.08 g	2.15 ± 0.67 ef	1.59 ± 0.08 cd	1.12 ± 0.13 e	290.34 ± 4.02 a	244.73 ± 2.27 b	1.29 ± 0.05 d	2.92 ± 0.11 a
	I:M 2:1	0.05 ± 0.01 i	2.94 ± 0.23 e	0.24 ± 0.01 g	0.18 ± 0.09 g	267.62 ± 1.87 b	234.59 ± 2.75 c	1.64 ± 0.33 ab	1.99 ± 0.18 d
	I:M 1:2	9.22 ± 0.45 d	15.35 ± 2.53 d	1.06 ± 0.11 e	0.79 ± 0.17 f	285.03 ± 2.16 a	232.87 ± 3.64 c	1.32 ± 0.11 cd	1.42 ± 0.17 ef
VD 90 °C	INU	75.21 ± 1.74 a	94.20 ± 4.35 a	0.71 ± 0.12 ef	0.14 ± 0.03 g	191.24 ± 1.54 g	195.87 ± 1.58 ef	1.12 ± 0.06 d	1.82 ± 0.25 d
	MALTO	0.39 ± 0.02 gh	11.53 ± 1.04 c	1.16 ± 0.17 de	0.89 ± 0.12 ef	298.68 ± 5.34 a	200.13 ± 2.28 ef	1.29 ± 0.03 c	1.35 ± 0.19 f
	I:M 2:1	75.07 ± 1.19 a	101.40 ± 4.93 a	0.71 ± 0.09 ef	0.25 ± 0.08 g	219.68 ± 3.74 e	188.92 ± 2.22 f	1.42 ± 0.05 c	1.76 ± 0.22 de
	I:M 1:2	47.12 ± 0.80 b	70.53 ± 3.66 b	0.24 ± 0.03 g	0.10 ± 0.02 g	181.80 ± 2.81 h	175.41 ± 3.64 f	0.85 ± 0.07 e	2.19 ± 0.11 c
**Duncan’s Multiple Range Test**									
Drying method	SD	0.94 C	1.57 C ^↑67.0%^	2.51 A	2.30 A ^↓8.4%^	235.38 BC	183.11 C ^↓22.2%^	1.61 A	2.04 A ^↑26.7%^
	FD	0.15 C	0.33 C ^↑120.0%^	1.78 B	1.73 B ^↓2.8%^	241.49 BC	229.76 A ^↓4.8%^	1.54 A	1.94 AB ^↑26.0%^
	VD 50 °C	0.33 C	0.90 C ^↑173%^	1.39 B	1.16 B ^↓16.5%^	253.61 B	213.33 B ^↓15.9%^	1.51 A	2.18 A ^↑44.4%^
	VD 70 °C	5.95 B	10.39 B ^↑74,6%^	1.03 BC	0.76 C ^↓26.2%^	271.71 A	231.17 A ^↓14.9%^	1.41 AB	2.00 A ^↑41.8%^
	VD 90 °C	49.45 A	69.42 A ^↑40.4%^	0.71 C	0.35 D ^↓50.7%^	222.85 C	190.08 C ^↓14.7%^	1.17 B	1.75 B ^↑49.6%^
Carrier agents	INU	18.26 A	29.45 A ^↑61.3%^	1.58 B	1.29 B ^↓18.4%^	215.38 C	192.62 C ^↓10.6%^	1.49 A	1.92 A ^↑28.9%^
	MALTO	0.41 D	4.80 C ^↑1070.7%^	1.83 A	1.65 A ^↓9.8%^	267.39 A	231.97 A ^↓13.2%^	1.41 AB	1.93 A ^↑36.9%^
	I:M 2:1	15.23 B	26.59 A ^↑74.6%^	1.30 C	1.06 C ^↓18.5%^	246.74 B	210.81 B ^↓14.6%^	1.55 A	2.08 A ^↑34.2%^
	I:M 1:2	11.56 C	17.77 B ^↑53.7%^	1.23 C	1.03 C ^↓16.3%^	250.52 B	202.55 BC ^↓19.1%^	1.33 B	1.99 A ^↑49.6%^

Data are shown as mean (*n* = 3) ± standard deviation; for each parameter tested, values with different letters differ significantly (Duncan’s test, *p* < 0.05); SD–spray drying; FD, freeze drying; VD, vacuum drying; INU, inulin; MALTO, maltodextrin; I:M, inulin:maltodextrin.
